# Impact of scaling up of kangaroo mother care on neonatal mortality among babies born with birth weight <2000 g in a district in southern India: a prospective cohort analysis

**DOI:** 10.1136/bmjph-2023-000349

**Published:** 2024-05-02

**Authors:** Tinku Thomas, Arin Kar, Suman P N Rao, Swaroop Narayana, Maryann Washington, Shashidhar Rao, Krishnamurthy Jayanna, Prabhu Deva Gowda, Mohan Harnahalli Lakkappa, Prem Mony

**Affiliations:** 1 Division of Epidemiology, Biostatistics & Population Health, St John's Research Institute, St John's Medical College, Bangalore, Karnataka, India; 2 Karnataka Health Promotion Trust, Bangalore, Karnataka, India; 3 Department of Neonatology, St John's Medical College Hospital, Bangalore, Karnataka, India; 4 Department of Health & Family Welfare, Government of Karnataka, Bangalore, Karnataka, India

**Keywords:** Public Health, Epidemiology, Community Health, Public Health Practice, Preventive Medicine

## Abstract

**Objective:**

To assess the impact of scaling up of kangaroo mother care (KMC) on neonatal mortality among babies born with birth weight <2000 g across an entire district in southern India.

**Design, setting and participants:**

Within an implementation research setting, analysis of a prospective birth cohort of babies with birth weight <2000 g born during March–December 2018 in Koppal district of Karnataka state, India, to estimate the incidence, risk and HRs of neonatal mortality associated with KMC.

**Intervention:**

Initiation and maintenance of KMC.

**Outcome measures:**

Neonatal mortality.

**Results:**

Among 23 667 live births, 1152 (4.9%) had birth weight <2000 g; the birth weight was <1500 g in 24% and <1000 g in 4%. Among them, 213 (18%, 95% CI 16% to 21%) babies died during the neonatal period, with 56% of the mortality occurring in the first 3 days of life and risk of mortality decreasing with higher birth weight. Overall, KMC was initiated in 816 (71%) babies; KMC-initiated babies had a substantially lower risk of neonatal mortality (risk ratio 0.07 (95% CI 0.05 to 0.09)). In a subset of 705 babies ‘eligible-for-KMC’ after exclusion of deaths, referrals or ‘terminal discharges’ (leaving against medical advice) in the first 3 days of life, and whose mother was a resident of the study area, 88% (95% CI 85% to 90%) were initiated on KMC. The RR of mortality among KMC-initiated babies remained low at 0.05 (95% CI 0.03 to 0.08) after adjusting for covariates and propensity-score adjusted analysis to address selection bias. Among 874 babies with follow-up data till 29 days of life, neonatal mortality rate was 24.4% (95% CI 21.6% to 27.3%); it was 6.4% (95% CI 4.7% to 8.6%) among KMC-initiated babies and 74.8% (95% CI 67.8% to 79.1%) among non-initiated babies (n=233).

**Conclusion:**

KMC implementation across a district was associated with substantial reduction in neonatal mortality. Scaling up KMC coverage across large geographies could facilitate achieving global child survival targets.

**WHAT IS ALREADY KNOWN ON THIS TOPIC**

- Previous studies have shown that kangaroo mother care (KMC), initiated after the newborn is stabilised, is a clinically efficacious intervention that can positively impact neonatal morbidity and mortality in controlled settings such as clinical trials. More recently, studies have also shown that this underused intervention can be scaled up across large geographies. In addition to this evidence from trials and implementation research, additional evidence of its impact on neonatal survival in real-life operational settings can boost the case for widespread coverage of KMC in small and ill neonates in district and subdistrict hospitals.

**WHAT THIS STUDY ADDS**

- In a field setting of an entire district in southern India, we quantified and compared the neonatal mortality in a cohort of babies born with birth weight <2000 g from across 90 public and private sector healthcare facilities or at home.
- Of 23 667 live births during a 10-month period in the study area, 1152 (4.9%) babies were born with birth weight <2000 g. Of these babies, 24% weighed <1500 g and 4% were <1000 g at birth. Overall, KMC was initiated in 816 (71%) of the babies. Scaled up KMC coverage in an implementation science mode was associated with neonatal mortality reduction by about 90% among KMC-initiated babies compared with non-initiated babies.

**HOW THIS STUDY MIGHT AFFECT RESEARCH, PRACTICE OR POLICY**

- The substantial association of initiation and maintenance of KMC on neonatal survival in real-life practice settings has implications for incorporating KMC into health policies and programmes aimed at improving child health. Attaining 2030 Sustainable Development Goal targets of improved child survival appears to be within reach.

## Introduction

 Globally, about half of under-5 child deaths are among newborns. Improving newborn survival is essential for achieving Sustainable Development Goal 3.2 (SDG-3.2). Achieving this goal would mean reducing neonatal mortality rate (NMR) to 12 or lower deaths per 1000 live births by 2030 globally.^1^ Preterm birth is now the leading direct cause of these neonatal deaths.^2^ Key interventions for improving neonatal survival include kangaroo mother care (KMC, defined as prolonged skin-to-skin care of newborn by mother/carer during day and night, plus exclusive breast feeding or breast milk feeding), antenatal corticosteroids for women presenting with preterm labour, treatments for neonatal respiratory distress, induced hypothermia and parenteral antibiotics for neonatal infections.^3^ In India, about 55% of under-5 deaths are neonatal deaths. At current rate of decline and after accounting for the likely stalling effect of the COVID-19 pandemic, India is unlikely to achieve the 2030 SDG-3 NMR target.^4^ In low-birthweight infants, KMC reduces neonatal mortality by 40% in clinical trial settings.^5^ Although KMC is one of five basic emergency newborn care ‘signal functions’, it is an underused intervention.

Translating the knowledge on effective interventions to achieve scale with commensurate health impact remains a critical challenge in global health, with ‘scaling up’ being defined as ‘deliberate efforts to increase the impact of successfully tested health innovations to benefit more people and to foster policy and programme development on a lasting basis’.^6^ Achieving scale in KMC coverage has been demonstrated recently,^7 8^ but evidence of its impact on neonatal survival in real-world settings is however lacking. The objective of our study was to assess the impact of scaling up of KMC on neonatal mortality among babies born with birth weight <2000 g across an entire district in southern India.

## Methods

The overall study methods,^9^ with details for Karnataka, India^10^ and the resulting intervention coverage of ‘any KMC’ (85%) and ‘effective KMC’ at discharge (53%),^8^ have been published earlier and are summarised below.

### Study design and population

We undertook a cohort study within an implementation research initiative^9^ that used a health services research design incorporating mixed-methods research.^11 12^ Our study site was Koppal district of Karnataka state in southern India, as part of a multicentric study with three sites in India and four sites in Ethiopia.^9^ Koppal district, with a population of 1.53 million, is located in the relatively underdeveloped northern region of Karnataka. Compared with the rest of the state, this region had poorer health indicators (33% higher crude birth rate, 19.5 vs 15; and 42% higher infant mortality rate, 27 vs 19) at the start of the study.^13^ Urbanisation rate was 18%, and institutional delivery rate was 95%, with 80% of them occurring in government hospitals. There was suboptimal coverage of emergency obstetric and newborn care facilities at the population-level as per United Nations Population Fund (UNFPA) norms.^14 15^ NMR was 24 deaths per 1000 live births in 2017.

Study participants were a cohort of live births born in Koppal district with a documented birth weight of <2000 g. The intervention period was from October 2016 to December 2018, and the analysis period was March–December 2018.

### Procedures

The state government of Karnataka partnered with two local institutions, St John’s Research Institute and Karnataka Health Promotion Trust, to implement the study. Three teams—programme learning, implementation support and evaluation—were constituted with an overall aim of reducing neonatal mortality via scaling up KMC as an intermediate outcome. The three teams helped with key tasks such as formative research, health facility-preparedness assessment and strengthening of care for small-and-ill babies. They also assisted with the capacity-building of healthcare personnel working in the community and health facilities through mentoring support for competency-based essential newborn care and care of small-and-ill babies in general and KMC in particular. In addition, an independent outcome assessment by research staff blinded to KMC exposure documented by the implementation teams was also undertaken. ‘Any KMC’ was defined as ‘prolonged skin-to-skin care of the baby by the mother/carer for as long as possible during day and night, and exclusive breast feeding or breast milk feeding’. ‘Effective KMC’ was defined as ‘skin-to-skin care for ≥8 hours and exclusive breast feeding in the 24-hour period before discharge’.^9^

Our implementation model had three components, namely: (1) Prefacility component activities aimed at high-quality birth weight screening of all births at the 90 (55 public and 45 private) health facilities (95%) and homes (5%) to identify newborns weighing <2000 g and enabling access to KMC-implementing facilities, (2) KMC-implementing facility component aimed at initiating and maintaining KMC for stable, in-born/out-born babies weighing <2000 g and (3) postfacility component of community-level activities aimed at continuing KMC after discharge. The study was carried out in two phases: phase I consisting of formative research and design of a KMC implementation model, followed by phase II consisting of roll-out for adoption and implementation across the entire district in stages.

At birth, all babies were classified as live births or still births. Stable babies were identified as ready for KMC while sick newborns were first stabilised and then considered for KMC. A stable newborn was defined as one whose vital functions (breathing and circulation) did not require continuous medical support and monitoring, and who did not experience rapid and unexpected deterioration regardless of intercurrent disease. Babies, once deemed stable by the primary care-giving doctor, were initiated on KMC and the carers and healthcare professionals were then supported to increase the number of hours of kangaroo care per day till discharge also to promote breast feeding. At the district levels and facility levels, resource mobilisation of personnel, infrastructure and drugs/equipment/supplies for appropriate clinical care for the newborn and arranging for amenities to ensure the comfort of the mother were undertaken from within the existing health system to ensure sustainability. To enhance quick uptake of KMC, mothers were provided with a KMC Kit that included a towel-wrap for the mother to facilitate kangaroo care, a KMC bag to hold baby, cap, socks, a breast milk collection cup and a paladai (traditional infant feeding cup).^16^

All live births with birth weight <2000 g were eligible for analysis. In addition, a subset of babies was further categorised as ‘eligible-for-KMC’ if the mother was a ‘resident of the study area’ and ‘after exclusion of deaths, referrals to a higher centre and terminal discharges(ie*,* leaving against medical advice) in the first 3 days of life’^8 9^

### Exposure assessment

The primary exposure was initiation of KMC. A brief case report form (CRF)—‘KMC Case-sheet’ —was used to capture details such as the timing and duration of kangaroo care and infant feeding (or parenteral nutrition) during each 24-hour period of hospitalisation till discharge. In addition, information on covariates relating to newborn (birth weight, place of birth, mode of delivery) and family (parental age, education and occupation; no of members in the household; no of living children; history of child death in the family; house ownership) was also collected.

### Outcome assessment

The primary outcome was neonatal mortality (defined as death in the first 28 days of life). Information on neonatal vital status (alive or dead) was collected from participating hospitals or during home visits till 29 days of life. NMR, defined as the number of deaths during the first 28 completed days of life per 1000 live births, was computed for babies followed up till 29 days of life.

### Sample size

For the primary objective of evaluation of 80% KMC coverage target, we had estimated a sample size of 310 newborns, with an absolute precision of ±5% and assuming that about 5% of newborns would be <2000 g and that a fifth of them would be lost to follow-up.^8 9^

### Statistical analysis

All continuous data are presented as median (quartile 1, quartile 3) and categorical data as number (%). All outcome estimates are presented as percentages with 95% CI. The association of neonatal mortality with KMC initiation adjusted for confounding characteristics, including birth weight, was examined using log-binomial regression, and the adjusted risk ratio (ARR) with 95% CI is reported. Further, to account for potential selection bias in initiation of KMC, we computed the propensity score for KMC initiation considering associated maternal and child characteristics—maternal education, birth order, place of delivery, mode of delivery and sex and birth weight of the baby. The propensity score was the probability of KMC initiation from the logistic regression and this score was considered as a covariate in the analysis of mortality for the propensity score-adjusted analysis. Additionally, sensitivity analysis was performed on two subgroups of babies: (1) ‘babies documented as healthy at the time of recruitment’ to exclude sick babies who were less likely to have been initiated on KMC and (2) babies who were followed up beyond 7 days of age, to exclude early neonatal deaths. The probability of survival in KMC initiated and uninitiated groups were compared using Kaplan-Meier plots and log-rank test. Further, this association was adjusted for confounding variables in the Cox proportional hazards regression model and adjusted HRs were calculated accordingly.

Since it was likely that heavier/healthier babies would more likely have been deemed eligible for KMC in the health facilities, we further examined these associations in the subset of ‘eligible-for-KMC’ babies. Statistical significance was considered at p<0.05 and all analyses were performed using STATA V.14 (StataCorp. 2015. Stata Statistical Software: Release 14, StataCorp).

### Patient and public involvement

Patients were not involved in setting the research question or the outcome measures. The mothers, carers, community members and health providers however contributed to iterations of the study design and implementation.

### Role of the funding source

The funders of the study had no role in study design, data collection/analysis/interpretation, or writing or submission of the report for publication. Three authors (TT, AK and PM) had full access to the data and had final responsibility for the decision to submit it for publication.

## Results

**Figure 1:**
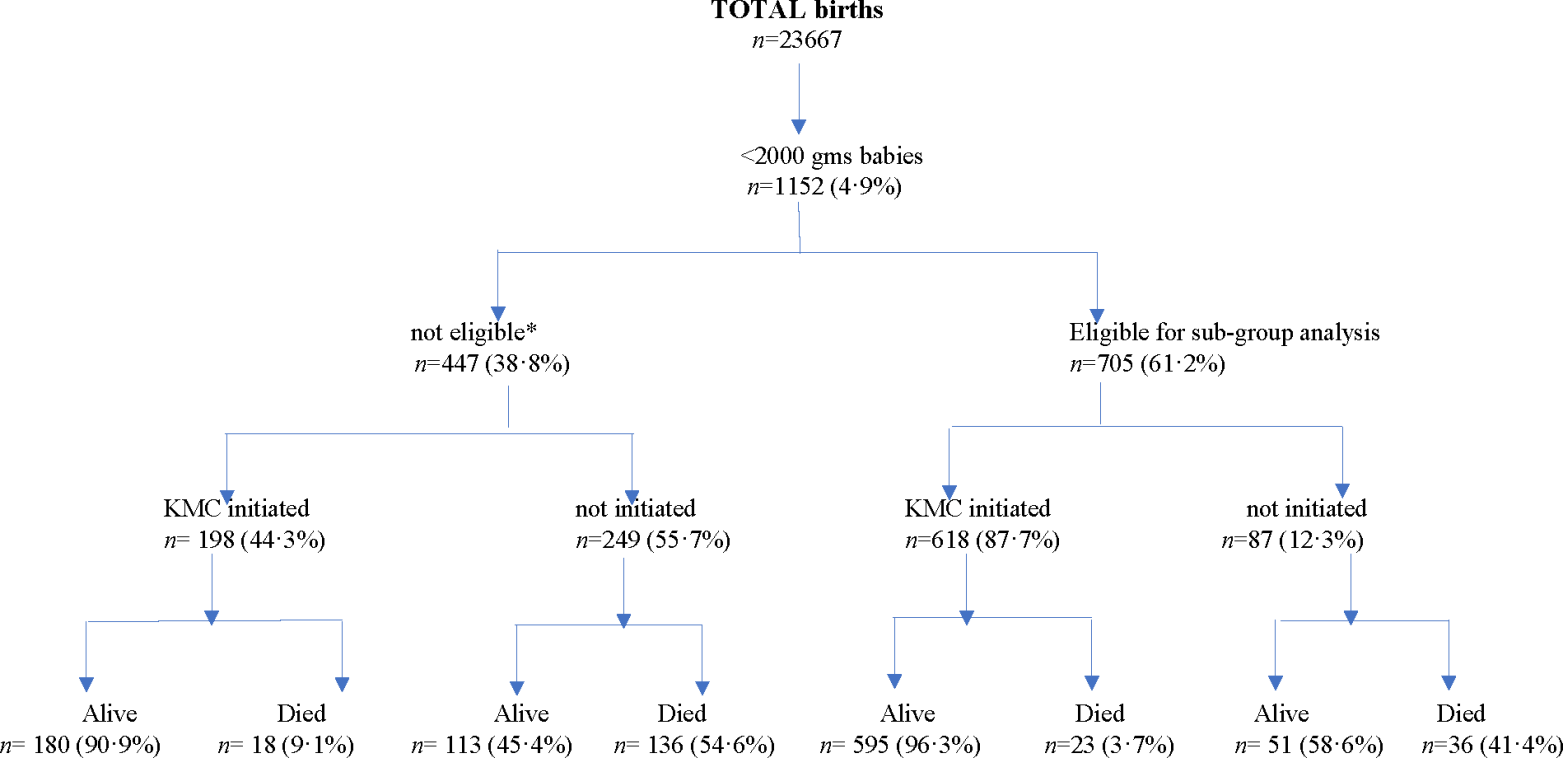
Flow chart of KMC initiation and vital status of all babies in Koppal district. *Number of babies not eligible for subgroup analysis: first 3 days’ exclusions (died (n=118), referred out to another hospital (n=210) or discharged against medical advice (n=25)); mothers: not resident in study area. KMC, kangaroo mother care.

There were a total of 23 667 live births in the study area; 1152 (4.9%) of these babies were born with birth weight <2000 g. In this study group, 1004 (87%) of the mothers were residents of the study area. Of the 1152 babies, 24% weighed below 1500 g and 4% were below 1000 g at birth. Among all babies, 210 babies were referred out of the study area to higher-level health facilities, 25 babies were ‘terminal discharges’ and 118 babies died within 3 days of birth (figure 1).

The overall neonatal mortality in the entire sample was 18% (95% CI 16% to 21%, n=213). Overall, KMC was initiated in 816 (71%) of the babies. The majority of deaths (n=172) occurred in the non-initiated babies such that 51% (172/336; 95% CI 61 to 72) of non-initiated babies died compared with 6% (41/816; 95% CI 4 to 8) of KMC-initiated babies.

The proportion of mortality decreased with birth weight (table 1). The mortality was higher in the non-initiated group at all levels of birth weight and there was no greater benefit of KMC on mortality in the 1000–1499 g birth weight group compared with the 1500–1999 g birth weight group (RR 0.10, 95% CI 0.06 to 0.16 vs RR=0.12, 95% CI 0.11 to 0.14). KMC was initiated in only seven babies in the <1000 g birthweight category.

**Table 1:** Birth weight category and mortality, n=1152

Birthweight category	Neonatal deaths					
	Total deaths		Deaths among KMC-initiated		Deaths among uninitiated	
	n	% (95% CI)	n/N_1_	% (95% CI)	n/N_2_	% (95% CI)
<1000 g (n=42)	27	64 (48 to 78)	0/7		27/35	77 (60 to 88)
1000–1499 g (n=232)	67	29 (23 to 35)	7/129	5 (3 to 11)	60/103	58 (48 to 67)
1500–1999 g (n=878)	119	14 (11 to 16)	34/680	5 (4 to 7)	85/198	43 (36 to 50

KMCkangaroo mother careN_2_no. of KMC un-initiated babiesnno. of deaths in the groupN_1_no. of KMC-initiated babies

Overall, the ARR for mortality in the KMC-initiated babies was 0.07 (95% CI 0.05 to 0.09), after adjusting for birth weight in logistic regression. Of the total 213 deaths, day-of-death information was available for 209 deaths, with 56% (118/209) occurring in the first 3 days of life.

**Figure 2:**
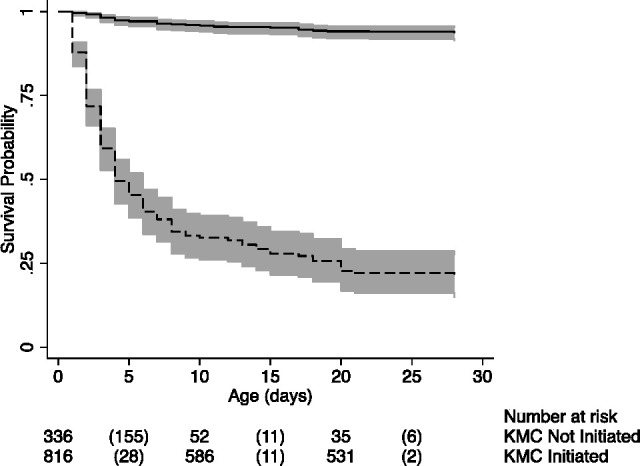
Kaplan-Meier estimates of neonatal survival by KMC initiation among all babies in Koppal district. Bold line represents KMC initiated group and dashed line represents KMC not-initiated group. Values in parenthesis represent mortality. KMC, kangaroo mother care.

The Kaplan-Meier plot of neonatal survival among all babies is shown in figure 2. The HR for mortality in the KMC-initiated group was 0.05 (95% CI 0.04 to 0.08), such that KMC-initiated babies had a 95% lower hazard of mortality compared with the uninitiated babies at any time within 28 days after birth, after adjusting for birth weight of the baby. The survival benefit in the initiated group was noted soon after birth.

The characteristics of the subset of 705 ‘eligible-for-KMC’ babies and their families are given in table 2. Among these babies, 88% (95% CI 85% to 90%) were initiated on KMC, of whom 38% were initiated within 24 hours, and 96% continued to receive KMC till discharge, with 64% babies having received ‘effective KMC’ at discharge. The median duration of KMC at discharge was 9 hours (Q1, Q3: 6, 13). About 95% of the babies for whom data was available 7 days postdischarge (n=511), continued receiving KMC. Overall, 54 babies (8%, 95% CI 7% to 11%) died during the neonatal period; neonatal mortality in the KMC-initiated group was 4% (95% CI 2% to 5%) vs 43% (95% CI 33% to 53%) in the uninitiated group.

**Table 2:** Characteristics of households, mothers and children in KMC eligible group, n=705

Characteristics*	Median (quartile 1, quartile 3)	No. (%)
Birth weight (g)	1780 (1580, 1900)	
Place of birth		
Home		25 (4)
Government facility		445 (64)
Private facility		234 (33)
Mode of delivery		
Normal		474 (80)
Caesarean		116 (20)
Age of mother (years)	23 (21, 26)	
Mother’s education		
None		132 (22)
Primary		125 (21)
Middle and high school		271 (46)
Above high school		63 (11)
Mother’s occupation		
Does not work for remuneration		376 (64)
Employed in formal/informal sector		59 (10)
Agriculture		152 (25)
Others		5 (1)
Number of living children		
0		312 (53)
1		185 (31)
≥2		95 (16)
History of child death in the family		
Yes		72 (12)
No		518 (88)
Age of father (years)	28 (26, 30)	
Father’s education		
None		152 (26)
Primary		109 (28)
Middle and high school		222 (38)
Above high school		103 (18)
Number of members in household	7 (5,9)	
Own house		
Yes		542 (92)
No		49 (8)

* sSample size varies from 586 to 705.

KMCkangaroo mother care

Table 3 shows the factors associated with neonatal mortality in the 705 babies. The ARR of mortality among KMC-initiated babies was 0.05 (95% CI 0.03 to 0.08) after adjusting for type of birthing facility, birth weight of baby, mode of delivery and history of a prior child death for the mother. When KMC initiation was replaced with effective KMC, that also remained significant, AR 0.05 (95% CI 0.02 to 0.16).

**Table 3:** Factors associated with neonatal mortality among eligible babies, n=705

	Unadjusted risk ratio (95% CI)	Adjusted risk ratio* (95% CI)
KMC initiation		
No (ref)	1	1
Yes	0.08 (0.05 to 0.13)	0.05 (0.03 to 0.08)
Mother’s education		
No education (ref)	1	
Primary	0.52 (0.26 to 1.05)	
Above primary	1.11 (0.59 to 2.12)	
Father’s education		
No education (ref)	1	
Primary	0.93 (0.44 to 1.98)	
Above primary	1.43 (0.72 to 2.85)	
Number of living children	1.09 (0.80 to 1.50)	
Birth order of the child	1.12 (0.87 to 1.44)	
Place of birth		
Government facility/home (ref)	1	1
Private health facility	0.68 (0.39 to 1.20)	0.62 (0.35 to 1.08)
Mode of delivery		
Normal (ref)	1	1
Caesarean	0.44 (0.16 to 1.19)	0.39 (0.14 to 1.05)
Prior history of child death in the family		
No (ref)	1	1
Yes	1.96 (0.98 to 4.93)	1.75 (0.89 to 3.45)

* n=587.

KMCkangaroo mother care

The ARR for age at KMC initiation was 1.06 (95% CI 0.99 to 1.14, p=0.082) such that the risk of mortality increased with delay in initiation of KMC. The median age of initiation of KMC was 2 days (Q1, Q3: 1, 6) in babies who survived vs 3 days (Q1, Q3: 2, 14) in babies who died. Neither place of birth nor mode of delivery interacted with initiation.

Data on breastfeeding initiation and time of initiation from birth were available in 585 (83%) babies eligible for the study. Breast feeding was initiated in 96% of the babies and the initiation happened within the first 24 hours for 64% of the babies. Among the 21 babies in whom breast feeding was not initiated, mortality was 64% vs 4% in initiated babies (OR 4.2, 95% CI 1.2 to 14.3). (This variable was not included in the multiple log binomial model analysis as the sample size was insufficient).

**Figure 3:**
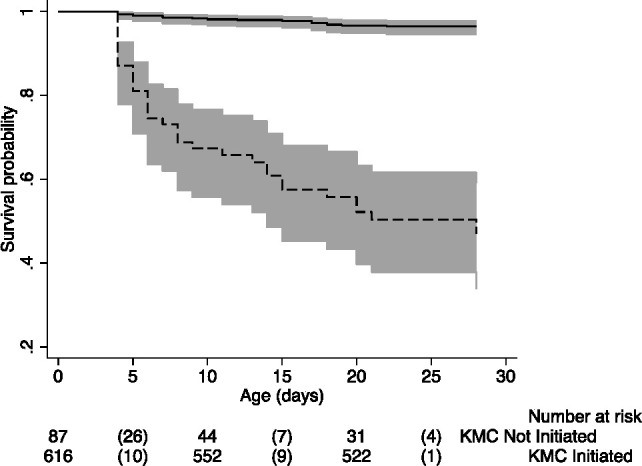
Kaplan-Meier plot of neonatal survival by KMC initiation among KMC eligible babies in Koppal district. Bold line represents KMC initiated group and dashed line represents KMC not-initiated group. Values in parenthesis represent mortality. KMC, kangaroo mother care.

The Kaplan-Meier plot of neonatal survival among babies after first 3 days’ exclusion is shown in figure 3. There was a significantly lower HR for mortality in KMC-initiated children after adjusting for birth weight in a Cox regression model (HR 0.06, 95% CI 0.03 to 0.11).

In the 587 babies who were considered for adjusted analysis based on availability of data on all covariates, 30 were sick at recruitment and of whom 14 (47%) were initiated on KMC.

### Sensitivity analysis

To correct for potential selection bias of KMC initiation, propensity score-adjusted analysis was performed and the RR of mortality continued to be low at 0.3 (95% CI 0.02 to 0.06). Among the 557 children who did not have any illness, 517 (92%) were initiated on KMC with a risk of mortality of 0.04 (95% CI 0.02 to 0.07). After excluding early neonatal deaths, among 213 babies followed up for at least 7 days, the risk of mortality was 0.08 (95% CI 0.03 to 0.18).

**Figure 4:**
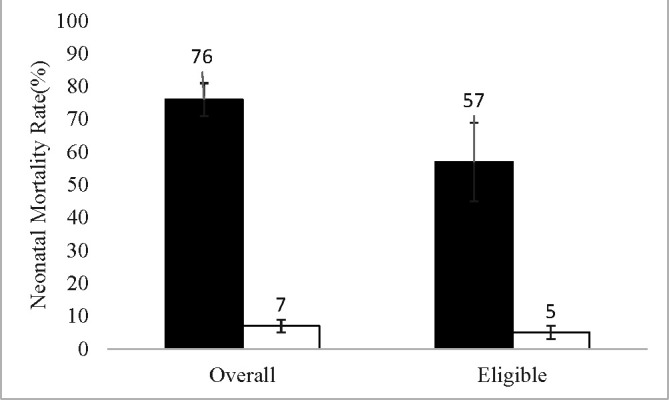
Neonatal mortality rates by eligibility and KMC initiation in Koppal district. Error bars indicate 95% confidence limits. The dark bar represents KMC initiated group and white bar represents KMC not-initiated group. KMC, kangaroo mother care.

Overall, the status at 29 days was known for 874 babies. The NMR was calculated to be 24.4% (95% CI 21.6% to 27.3%). NMR among KMC-initiated babies (n=641) was 6.4% (95% CI 4.7% to 8.6%) compared with 74.8% (95% CI 67.8% to 79.1%) among babies not initiated on KMC (n=233). Among these 874 babies, 602 were KMC-eligible babies and the NMR was 9.8% (95% CI 7.7% to 12.5%). The NMR was 4.3% (95% CI 2.8% to 6.4%) in KMC-initiated group (n=538) and 56.2% (95% CI 43.7% to 68.0%) in the uninitiated group (n=64). NMRs by KMC initiation and eligibility are shown in figure 4. There was a significant association between KMC initiation and follow-up until 29 days: 79% of the KMC-initiated babies were followed up while only 69% babies were followed up in the uninitiated group (p=0.001). However, the birth weights of the babies followed up until 29 days were comparable to that of the babies not followed up (1643±10 vs 1601±18 g).

## Discussion

KMC intervention was found to be associated with a 90% reduction in neonatal mortality for babies with birth weight less than 2000 g in our study setting. To our knowledge, this is the first study to detect such an association of KMC on neonatal survival in a real-life setting with scale-up of the intervention across an entire district.

The first question is whether the observation is real. We believe our findings are probably real. While the ‘healthy-entrant effect’ selection bias was likely operational in the subset of babies included in the analysis after exclusion of first 3 days’ outcomes (owing to birth weight or the clinical condition of the newborn being a determinant of initiation of KMC), this was likely not a factor in the analysis of the full set of all babies born in the district. Attrition bias, owing to significantly differential rates of follow-up of KMC-initiated and uninitiated babies noted in our study, was again probably operational in the subset of ‘KMC-eligible’ babies; but we noticed that mean birth weights did not differ substantially in the two subgroups.^17^

Information bias was not likely to have affected the study findings.^18^ Ascertainment of vital events (births or deaths) was by a standard method of data collection by research staff who visited health facilities and communities and were blinded to the KMC data. Suboptimal reporting of deaths, if any, was likely to have been higher in the non-initiated, non-followed up group, indicating a greater beneficial effect of KMC. Exposure ascertainment was captured by a specially designed CRF and corroborated by independent research staff during home visits. While it has been shown in a device-validation study that the duration of kangaroo care may be misreported by mothers/carers,^19^ the fact of KMC initiation itself was less likely to be mis-reported,^19^ as noted in a substudy.^20^

Reverse causality and circular reasoning remain a concern in observational studies. The sensitivity analysis performed is likely to have mitigated this concern substantially. Further, the beneficial effect of KMC on neonatal survival was seen in the entire group of newborns as well as in the subset of ‘eligible-for-KMC’ newborns among whom some received KMC and some did not—this subset of babies with two subgroups (after 3 days’ exclusions) is closest to a proper counterfactual within a real-life setting as ours.

The next question is whether this scale of impact of KMC on newborn survival is possible. In addition to KMC, cointerventions such as refresher training of healthcare workers, emphasis on documentation and quality improvement initiatives were also likely to have contributed to the observed effect. On-site mentoring to reinforce essential newborn care, infection control and supportive supervision, etc as a package have been documented to benefit health workers’ knowledge and skills earlier.^21^ Use of case sheet has been shown to help improve the adoption and quality of documentation for neonatal care.^22^ Supportive supervision, which is managerial support for healthcare workers through periodic visits from higher-level hospitals to peripheral facilities, may have contributed indirectly to support quality of care and improve provider motivation through non-punitive review of practices and mentoring.^21 23^ ‘Hawthorne effect’ is also a likely explanation; engaging the workers in a ‘mission mode’ and saturation of 100% of health facilities and communities across the entire district for adoption and maintenance of KMC could have contributed to the observed effect.^24^ Continuous quality improvement strategies that engaged facility and health system leaders to identify root/proximal causes amenable to change and subsequently employing local strategies for addressing those causes could also have contributed.^25^

The effect size noted in our implementation research setting was higher than that seen for various interventions in clinical trial settings in low-income and middle-income countries.^26^ This was likely because the introduction of KMC acted as a gateway for the establishment of care for small-and-ill babies in our setting.^27^

Certain key issues and limitations merit further discussion. One key limitation of our study was the inability to tease out the effect due to KMC per se as an intervention apart from the effects due to the quality improvement strategies that were also part of the implementation programme. Further, though we adjusted for birth weight and several known risk factors, the role of other unknown or unmeasured confounding factors (such as newborn feeding, health system factors) affecting KMC as well as neonatal survival cannot be ruled out. A substantial portion of the beneficial effect was however most likely due to the kangaroo care itself.^27^ Reverse causality needs to be carefully considered in real-life effectiveness studies; our sensitivity analysis might have ameliorated this concern and contributed to supporting the robustness of the conclusion regarding causal inference. In addition, the statistical adjustment of propensity score balanced the conditional receipt of KMC by a group of newborns on observed baseline covariates such that the groups who received or did not receive KMC were comparable. Since underlying/immediate causes of death were not accurately identified for the newborn deaths, it was not possible to specify the relative impact of KMC on different causes of deaths in relation to others. In spite of these limitations, the role of ‘implementation science’ cannot be overlooked—performance implementation that was evidence based, contextual, stage based, with implementation teams working to ensure fidelity of KMC roll-out, along with government leadership and health workers’ commitment.^28^

Our study has shown that it was possible to impact neonatal survival (especially among babies with birth weight 1000–2000 g) in a relatively underdeveloped district; this should hence be scalable across the country with NMRs varying 4–5 times across states and 7–8 times across districts.^29^

In conclusion, initiation and maintenance of KMC was associated with a substantial impact on improving neonatal survival as evidenced in this implementation research study across an entire district with 90 health facilities in a state in southern India. It is a scalable and efficacious intervention that now offers hope of bringing down neonatal and child mortality to targeted levels by 2030 within reach.

## Data Availability

Data are available on reasonable request.
